# Whole-genome sequencing data from long-term serial passaging of 11 SARS-CoV-2 isolates

**DOI:** 10.1128/mra.01090-25

**Published:** 2025-11-06

**Authors:** Charles S. P. Foster, Gregory J. Walker, Tyra Jean, Maureen Wong, Sonia R. Isaacs, Yonghui Lyu, William Rawlinson

**Affiliations:** 1 Serology and Virology Division (SAViD), NSW Health Pathology, Prince of Wales Hospital, Sydney, New South Wales, Australia; 2 School of Biomedical Sciences, Faculty of Medicine and Health, University of New South Wales, Sydney, New South Wales, Australia; 3 School of Clinical Medicine, Faculty of Medicine and Health, University of New South Wales, Sydney, New South Wales, Australia; 4 School of Biotechnology and Biomolecular Sciences, Faculty of Science, University of New South Wales, Sydney, New South Wales, Australia; DOE Joint Genome Institute, Berkeley, California, USA

**Keywords:** SARS-CoV-2, whole-genome sequencing, serial passaging

## Abstract

Serial passaging is useful for investigating the evolutionary trajectory of viruses *in vitro*, allowing comparison with evolutionary trends in the real-world human population. We announce here a 2020–2023 longitudinal data set comprising amplicon-based whole-genome sequencing from 11 severe acute respiratory syndrome coronavirus 2 (SARS-CoV-2) isolates carried through a range of 33–100 passages.

## ANNOUNCEMENT

We recently investigated the accumulation of mutations in SARS-CoV-2 (*Coronaviridae: Betacoronavirus*) in a long-term serial passaging study, scanning for any instances of convergent evolution among passage lines and compared to global clinical isolates (1). Eleven isolates of SARS-CoV-2 were selected, representing nine distinct SARS-CoV-2 lineages, ranging from earlier (A.2.2) to later (Omicron: BA.1) in the coronavirus disease 2019 (COVID-19) pandemic, prioritizing variants of concern and variants under investigation, as designated by the World Health Organization. Isolates originated from remnant human nasopharyngeal swabs collected during routine diagnostic testing in the Southeastern Sydney Local Health District, NSW, Australia. After spin-filtration of swabs in virus-transport media, 100 µL of virus-containing flow-through was used to inoculate Vero E6 cells (ECACC #85020206) maintained in Gibco Minimum Essential Medium (Thermo Fisher, Massachusetts, USA) supplemented with 10% fetal bovine serum and 1× penicillin-streptomycin-glutamine (MEM-10), and incubated at 37°C, 5% CO_2_. Each passage interval spanned 3–4 days until cytopathic effect was observed, then harvested cultured virus inoculated the next passage, described fully in reference 1. The goal was a minimum of 33 serial passages per passage line, with earlier passage lines continued further (e.g., B.1.319: 100 passages).

**Fig 1:**
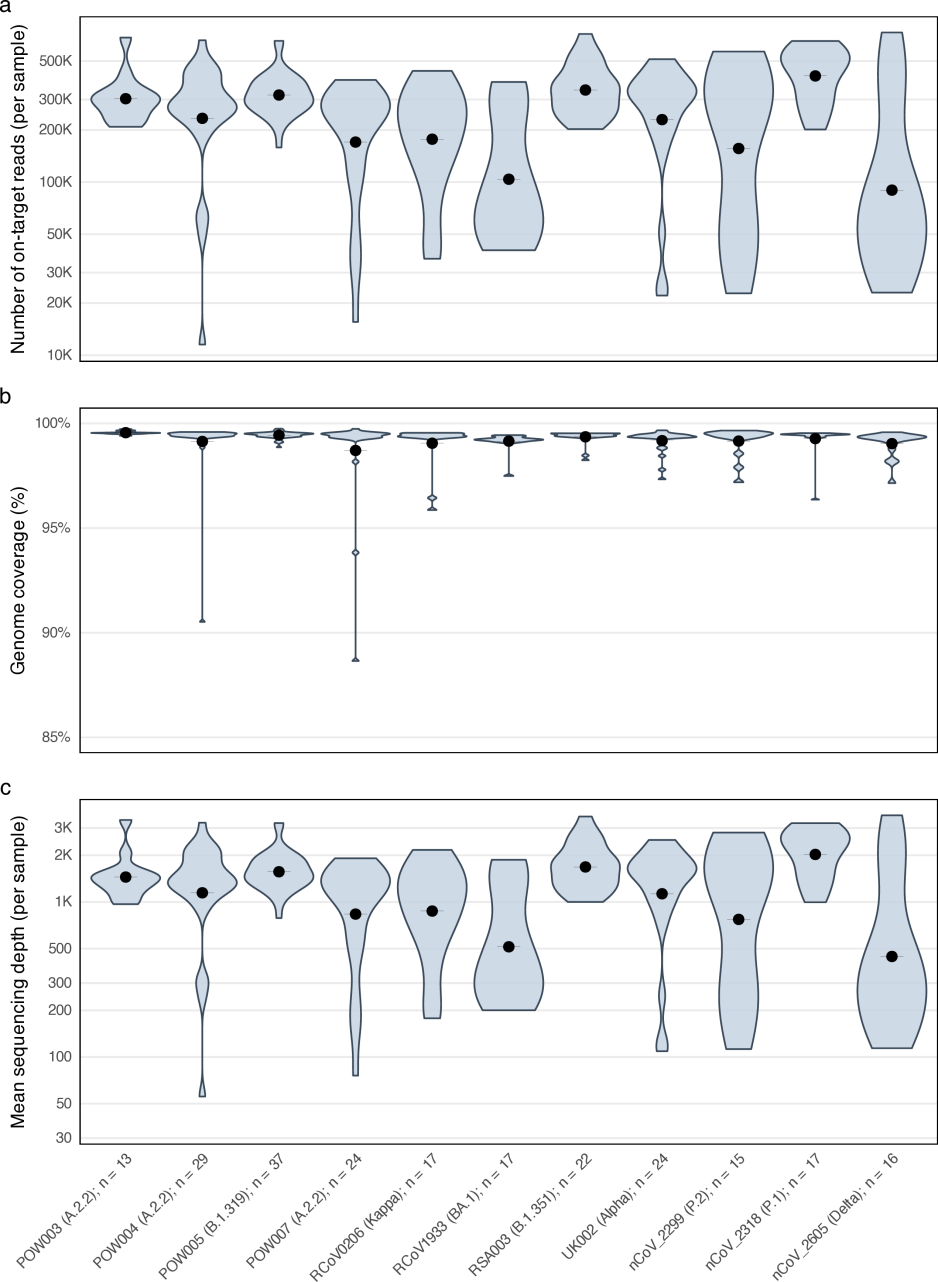
Sequencing metrics for serially passaged SARS-CoV-2 isolates, summarized by passage line. Violin plots show per-sample distributions; black dots/crossbars indicate the mean. (**a**) Number of on-target reads per sample (log10 scale). (**b**) Genome coverage (% of genome covered, ≥10× ). (**c**) Mean sequencing depth per sample (log10 scale). Note: these statistics are given excluding one outlier poorly sequenced sample in the RCoV0206 passage line (SRA accession no. SRR26083382). All metrics were estimated using the default parameters of an in-house bioinformatics pipeline (4), with low quality sequencing reads and residual sequencing adapters removed using fastp v.0.23.2 (5), read mapping against the SARS-CoV-2 reference genome (NC_045512.2: 29903 nt, ~38% GC) performed using bwa mem v.0.7.17-r1188 (6), soft clipping of amplicon primer regions using ivar v.1.3.1 (7), and genome coverage estimation using bedtools v.2.30.0 (8). Labels on the *x*-axis refer to in-house identifiers for passage lines, matching those given to the sets of sequencing reads uploaded to National Center for Biotechnology Information’s Sequence Read Archive (PRJNA1018257).

Our whole-genome sequencing strategy was (where possible) to sequence the original clinical isolate (passage 0), passages 1–6, then every third passage onward. Some passage 0 samples were from urgent clinical cases sequenced via rapid Oxford Nanopore Technology approaches, but this Announcement focuses on the remaining majority short-read sequencing data, generated as described previously (1, 2). Briefly, nucleic acids were extracted using the MagNA Pure 96 system (Roche Diagnostics, Mannheim, Germany); RNA extracts were reverse-transcribed with the SuperScript IV VILO Master Mix (Thermo Fisher); cDNA was amplified using 1,200 nt tiled amplicons per the “Midnight” protocol (3); amplicon products were prepared for short-read sequencing using the Illumina DNA Prep Kit (Illumina, San Diego, CA, USA); and 150 bp paired-end sequencing was conducted using an Illumina Miseq (Reagent Kit v.2). Each of these steps was conducted as per the respective manufacturers’ protocols. The resulting 231 sets of paired-end sequencing reads were analyzed using an in-house bioinformatics pipeline with default parameters (4), as described in reference 1, yielding a mean genome coverage of >99%, sequencing depth of ~1,372×, and ~279,000 (range: 11,533–731,494) reads per sample, with relative consistency among passage lines (Fig. 1).

Following the trajectory of mutations within these data longitudinally reveals important insights into pathogen evolution and broader evolutionary mechanisms, such as convergent evolution, adaptation, and genetic drift (1). For example, many mutations that arose *de novo* throughout serial passaging are known to drive changes in cell entry mechanisms (9), especially in Vero E6 cells (10), and are linked to clinically significant phenomena like altered therapeutic effectiveness (11). Additionally, we demonstrated that SARS-CoV-2 can be passaged up to 100 times without any obvious signs of impaired fitness or attenuation (1). We anticipate that these sequencing data will be a useful resource for the microbiology community interested in SARS-CoV-2 evolution and experimental evolution more generally.

## Data Availability

The consensus genomes for all clinical isolates that were subsequently taken through serial passaging are available through the Global Initiative on Sharing All Influenza Data (GISAID), collated under the EPI_SET identifier EPI_SET_250922xh (https://doi.org/10.55876/gis8.250922xh). All sequencing reads are available from the National Center for Biotechnology Information’s Sequence Read Archive under accession number PRJNA1018257 (https://www.ncbi.nlm.nih.gov/bioproject/PRJNA1018257). The pipeline used to process and analyze the reads is available at https://github.com/charlesfoster/covid-illumina-snakemake.
